# Supplementary material for: Patterns of Intron Gain and Loss in Fungi
Source: PLoS Biol. 2004 Nov 30;2(12):e422. doi: 10.1371/journal.pbio.0020422 (PMC532390; doi:10.1371/journal.pbio.0020422)
Supplement: Table S1 — Also available at http://genes.mit.edu/NielsenEtAl/. (4.3 MB ZIP). [file pbio.0020422.st001.zip › NielsenEtAl/html/882.html]

AN4382.1.NCU04309.1.MG02303.1.FG10322.1


```
 CLUSTAL W (1.82) Multiple Sequence Alignments - Introns Inserted


Sequence 1: NCU04309.1	244 aa
Sequence 2: FG10322.1	236 aa
Sequence 3: AN4382.1	234 aa
Sequence 4: MG02303.1	236 aa
Alignment Length: 248 aa
Number Identitical Residues: 79 aa
Alignment Score (without introns) 4428


MG02303.1 	MDPQEVTGLAKKVWA1RYKLAEPTKRIL~IGIAGIPGAG~KTTLARRVVDELKAIQLAED
NCU04309.1	MEEQ-MDRLVERAWD~KFLDTPTDQRLL1IAIAGIPGSG1KTTLSQILARRLNILHATEY
FG10322.1 	MEDQ-VERLVEKAWK~KYEETPKDKRLL1IGVAGIPGSG1KTTFSQIITNRINARALSSD
AN4382.1  	MESQ-VARLVDKIWE1KFRTTPENARLL~IAVSGIPGSG~KTELAITMARRINEKHGAQN
          	*: * :  *..: *  ::  :    *:* *.::****:* ** ::  :. .::    :. 

MG02303.1 	G--TADGEFVTDIPMDGYHLTRAQLAAMPDPEMAIHRRGAAFTFDGEGFLALLQKLSLPV
NCU04309.1	PLASMSPDFATALPMDGFHLTRAQLSAMPDPELAHARRGAEFTFDGQGFYELVKELRKPV
FG10322.1 	P---SSPPPATFVPMDGFHLTRAALSAMPDPDNAHFRRGAAFTFDAPKFHALVQSLSKTP
AN4382.1  	G-----DLIAAAIPMDGYHLTRAQLAQMPDPEYAAARRGAAFTFDGEKFLALVRALREPL
          	         .: :****:***** *: ****: *  **** ****.  *  *:: *  . 

MG02303.1 	EEDSGAQGTVTIYAPSFDHAVKDPVADSIPISPKMRIVIIEGNY~LALDREPWKSAASLL
NCU04309.1	VTTATRT---TVWAPSFDHALKDPVEKGIEVGPEMRVVVFEGNY1LLLDQKPWSDAAKLM
FG10322.1 	ISSEP------ILAPSFDHALKDPRDDDIVVKPEHRVVVLEGNY~LALDQDVWRDAAKFL
AN4382.1  	TPKTQT-----LHAPSFDHAVKDPVDNDIPIAAARRVIFFEGNY~LSLNKEPWSSAAKLM
          	           : *******:***  ..* : .  *::.:**** * *::. * .**.::

MG02303.1 	DEIWFANVDREVARERLAKRHVEAGIVPDEEAARERIRTTDFLNADDIEQNLLP--VSER
NCU04309.1	DLKFFVRVPFPVARKRLVKRHLAAGIAATEEEADKRAVENDLVNGALIEELLREDEVDEV
FG10322.1 	DEVWFVEVDFEVARKRLRERHVRAGIVKDLEEGDRRAMESDLVNGKEIVDFRLK--VDEV
AN4382.1  	DELWFVDVDFDTARQRLVKRHVKAGIAKDEADAEKRADENDLVNGREIVDCRLD--VQEI
          	*  :*. *   .**:** :**: ***.     . .*   .*::*.  * :      *.* 

MG02303.1 	VRCG--------
NCU04309.1	VESLEDGKWVHT
FG10322.1 	IQSREDGSWVHK
AN4382.1  	IRSSYDPKWED-
          	:..  . .
```
